# Phylogenetic analysis and susceptibility to antibiotics and phytogenics of *Riemerella anatipestifer* strains isolated in Poland

**DOI:** 10.2478/jvetres-2026-0019

**Published:** 2026-03-30

**Authors:** Jan Tyc, Agnieszka Kalwasińska, Monika Olszewska-Tomczyk, Grzegorz Woźniakowski

**Affiliations:** Department of Infectious and Invasive Diseases and Veterinary Administration, Institute of Veterinary Medicine, Department of Environmental Microbiology and Biotechnology, Institute of Biology, Faculty of Biological and Veterinary Sciences, Nicolaus Copernicus University in Toruń, 87-100 Toruń, Poland; Department of Environmental Microbiology and Biotechnology, Institute of Biology, Faculty of Biological and Veterinary Sciences, Nicolaus Copernicus University in Toruń, 87-100 Toruń, Poland

**Keywords:** antimicrobial resistance, phylogenetic analysis, phytogenics, poultry, *Riemerella anatipestifer*

## Abstract

**Introduction:**

*Riemerella anatipestifer* (RA) is a causative agent of high-mortality *septicaemia anserum exsudativa*, which inflicts large economic losses on poultry farming. The growing concern over antibiotic resistance and imposed limitations on these drugs’ use in animals prompt the search for antibiotic alternatives. This study characterises the phylogenetic relationship of Polish RA strains and assesses their susceptibility to antibiotics and *trans*-cinnamaldehyde, geraniol, carvacrol and eugenol as antibiotic alternatives.

**Material and Methods:**

A total of 24 provided strains were confirmed by RA-specific PCR. A phylogenetic tree was generated with partial 16S ribosomal RNA sequences, also including *Riemerella*-type strains obtained from GenBank. Antibiotic susceptibility was determined by the Kirby–Bauer method and phytogenic susceptibility by the minimum inhibitory concentration (MIC) method.

**Results:**

The majority of the strains clustered together, and only one, which clustered with the RA type strain, was an outlier. The widest inhibition-zone diameters were obtained for cephalexin, ceftazidime and cefuroxime, while more than 70% of the strains showed no visible zone of inhibition under the action of enrofloxacin, clindamycin, erythromycin or streptomycin. The most effective phytogenic was *trans*-cinnamaldehyde, with the minimum concentrations inhibitory and bactericidal to 90% of isolates being only 75 μg/mL.

**Conclusion:**

The RA strains showed relatively low genetic diversity. Despite this, antimicrobial resistance varied significantly between them. Continuous monitoring of the resistance pattern is necessary. Interpretive criteria for RA susceptibility testing not having yet been standardised, existing research is inconclusive. Some phytogenics are promising alternatives to antibiotics; however, more pharmacokinetics and pharmacodynamics data are needed.

## Introduction

*Riemerella anatipestifer* (RA) is a gram-negative, rod-shaped, non-motile, non-spore-forming, catalase- and oxidase-positive bacterium belonging to the *Weeksellaceae* family. On blood agar, RA usually grows in 24–48 hours as 1–2 mm-diameter, convex, entire, transparent, glistening and butyrous colonies (25). It was described for the first time in 1904 by Riemer as an aetiologic agent of avian diphtheria in geese (22). Since then, it has been reclassified several times, from the genera *Pfeiferella, Moraxella* and *Pasteurella*; however, its phylogenetic relationship with other members of the *Weeksellaceae* family strongly suggested its separateness, and the *Riemerella* genus was proposed in 1993 as a tribute to Riemer’s discovery (27). Within the genus, two more species have been described, namely *R. columbina* (36) and *R. columbipharyngis* (23), primarily found in pigeons. *Riemerella anatipestifer* is a causative agent of the condition called *septicaemia anserum exsudativa*, also referred to as duck septicaemia, riemerellosis or infectious serositis. This disease is responsible for high mortality rates and large economic losses in various poultry species, especially in duck and goose flocks, and less frequently in turkey and chicken flocks (1). The disease form usually occurs as acute or chronic septicaemia with polyserositis, perihepatitis, pericarditis, pneumonia, airsacculitis or meningitis, with possible bone marrow and hock joint involvement resulting in locomotor symptoms. Young birds of 1–8 weeks of age are regarded as more susceptible to the acute form of infection. Ducklings, which are usually most susceptible until the end of the fifth week of life, usually die 1–2 days after clinical symptoms occur, if not treated (25). In general, waterfowl are more susceptible to the acute form of the disease, whereas in *Galliformes*, it presents rather as a localised form, characterised by respiratory, neurological and/or locomotor symptoms. Riemerellosis is also identified in wild geese and many wild *Anatidae* family species, including the mallard duck, northern pintail duck and spot-billed duck. Free-living birds can transmit RA infection to commercial flocks, as well as spread it to areas free from riemerellosis before succumbing to the disease, which is a very frequent outcome (4). For this reason, it is important to monitor the prevalence of riemerellosis, especially in countries with high poultry production, as well as provide proper biosecurity, to avoid disease outbreaks in areas of higher risk.

Poland is the biggest poultry producer in the European Union. According to the Food and Agriculture Organization of the United Nations, 2.28 million tonnes of chicken meat were produced in Poland in 2023, accounting for more than 20% of the EU’s total production (10). Maintenance of this production level is necessary to provide a sufficient amount of the product, as poultry is one of the most commonly consumed types of meat, with 16.47 kg consumed per capita in the EU annually (20). To achieve this, biosecurity measures must be followed; otherwise, infectious agents such as RA can easily spread into the environment and between farms, leading to high mortality, decreased growth rates and severe economic losses. In cases of bacterial disease outbreaks on farms, antimicrobial therapy is typically implemented; however, growing antibiotic resistance hinders the effectiveness of empirical antimicrobial treatment and antibiotic therapy in general. What complicates the problem are the tough restrictions on and close monitoring of antibiotic use in veterinary medicine (9), especially in livestock, which are maintained because of concerns over overuse and the potential risk of transmitting resistant pathogens to humans (18). The World Health Organization has published a list of medically important antimicrobials. To mitigate antimicrobial resistance, the use of some listed drugs by veterinarians should be critically restricted (as is use of the third and fourth generations of cephalosporins, quinolones and polymyxins), and the use of others may even be authorised only in human medicine (37). To restore therapeutic effectiveness and not exacerbate resistance, alternatives to antibiotics have been sought, among which essential oils and phytogenic feed additives (PFAs) based on them are of great interest (2). When using PFAs instead of antibiotics in situations needing antimicrobial activity, advantages to these preparations are seen, including anti-inflammatory, antioxidant, immunomodulatory and even growth-promoting properties (12). The relevant active substances can be derived from essential oils extracted from plants, particularly from genera such as *Origanum, Cinnamomum, Syzygium, Thymus* or *Rosa*. Among the most commonly utilised substances are *trans*-cinnamaldehyde, carvacrol, eugenol and geraniol. The use of these compounds is well documented in veterinary medicine, especially as feed additives (38).

Despite the pre-eminence of Poland as a poultry producer in Europe, there are not enough current data about the occurrence of RA in Polish poultry flocks, the phylogenetic relationship of Polish RA strains, their antibiotic susceptibility, or possible efficient anti-RA alternatives to antibiotics. This article aims to assess a selection of RA strains’ phylogenetic relationships and to provide the most current information on their susceptibility to antibiotics and phytogenics.

## Material and Methods

### Strain origin, sampling and DNA isolation

Bacterial strains were obtained in 2023 and 2024 at QLS-LAB Veterinary Diagnostic Laboratory, located in Żuromin, Poland, and were provided for further analysis. Strains were isolated from organ swabs collected during regular veterinary diagnostic procedures in commercial poultry flocks to identify the aetiological agent of a disease; therefore, approval from the Local Ethics Committee was not mandatory. A total of 24 strains were examined (10 isolated from turkeys, 12 from chickens, 1 from ducks and 1 from geese). Detailed information about the strain’s origin is presented in Table 1. Strains were isolated on Columbia agar with 5% defibrinated sheep’s blood (Oxoid, Basingstoke, UK), which was incubated in a candle jar for 48 h at 37°C. All the strains were tested for catalase and oxidase production and then Gram stained. A single colony of each strain was picked and incubated in BHI broth (Becton Dickinson, Franklin Lakes, NJ, USA) for 24 h at 37°C and then transferred to a sterile cryotube with glycerol solution and stored at –80°C for further analysis.

**Table 1.** *Riemerella anatipestifer* strain description and origin

Strain number	Poultry flock location (voivodeship)	Poultry species	Date of strain isolation	Anatomical site
910/24	Warmińsko-mazurskie	Turkey	03/09/2024	Trachea
899/24	Mazowieckie	Turkey	30/08/2024	Brain
767/24	Mazowieckie	Chicken	30/07/2024	Lungs
750/24	Świętokrzyskie	Chicken	27/07/2024	Trachea
660/24	Kujawsko-pomorskie	Goose	27/07/2024	Brain
596/24	Mazowieckie	Chicken	03/06/2024	Trachea
563/24	Mazowieckie	Chicken	30/05/2024	Lungs
503/24	Warmińsko-mazurskie	Turkey	15/05/2024	Trachea
369/24	Mazowieckie	Chicken	06/04/2024	Trachea
342/24	Pomorskie	Chicken	25/03/2024	Infraorbital sinus
337/24	Mazowieckie	Chicken	22/03/2024	Hock joint
272/24	Mazowieckie	Chicken	05/03/2024	Hock joint
1223/23	Mazowieckie	Duck	27/10/2023	Hock joint
1171/23	Mazowieckie	Chicken	14/10/2023	Infraorbital sinus
1034/23	Podlaskie	Turkey	13/09/2023	Trachea
984/23	Kujawsko-pomorskie	Chicken	30/08/2023	Trachea
833/23	Mazowieckie	Turkey	29/07/2023	Trachea
784/23	Warmińsko-mazurskie	Turkey	26/07/2023	Trachea
780/23	Warmińsko-mazurskie	Turkey	26/07/2023	Trachea
761/23	Warmińsko-mazurskie	Turkey	17/07/2023	Trachea
655/23	Mazowieckie	Chicken	23/06/2023	Lungs
310/23	Mazowieckie	Chicken	19/03/2023	Trachea
257/23	Mazowieckie	Turkey	06/03/2023	Trachea
152/23	Mazowieckie	Turkey	14/02/2023	Infraorbital sinus

### Isolation of DNA and species identification with PCR

To restore the bacteria, a loopful of thawed cryopreserved bacterial suspension was streaked onto plates with Columbia agar with 5% defibrinated sheep blood, and the plates were incubated for 48 h in a candle jar. Subsequently, a few single colonies were picked and transferred to 1 mL of sterile 0.85% saline, and bacterial DNA was isolated using a spin column–based nucleic acid purification method (A&A Biotechnology, Gdańsk, Poland), according to the manufacturer’s instructions. The final volume of purified DNA was 100 μL dissolved in Tris buffer. The quality and quantity of DNA were evaluated using the NanoDrop One UV-Vis spectrophotometer (Thermo Fisher Scientific, Wilmington, DE, USA). After extraction, samples were stored at -80°C until further analysis. For the identification of RA by PCR, the RA-specific primers were used which were described and validated by Rubbenstroth *et al*. (24). The PCR reaction mixture was composed of 1 μL of sample, 2.5 μL of 10× buffer, 0.5 mM of dNTPs, 0.5 mM of the RA L-17 (5ʹ-TAG CAT CTC TTG GAT TCC CTTC-3ʹ)and RA R-354 (5ʹ-CCA GTT TTT AAC CAC CAT TAC CC-3ʹ) primers, 1 U of Taq DNA polymerase, 1.5 mM of MgCl_2_ solution and 3 μL of KU buffer (all from A&A Biotechnology) in a final volume of 25 μL. The reactions were performed on a T100 Thermal Cycler (Bio-Rad Laboratories, Hercules, CA, USA) with the following set-up: initial denaturation at 95°C for 3 min; 35 cycles of denaturation at 95°C for 15 s, primer annealing at 55°C for 45 s and elongation at 72°C for 1 min; and a final elongation at 72°C for 10 min. The size of the PCR product was 338 base pairs (bp). The PCR products were quality checked by 1.5% agarose gel electrophoresis using a Mupid-One system (Advance, Tokyo, Japan) and visualised by SimplySafe staining (EURx, Gdańsk, Poland) under an ultraviolet transilluminator. The same PCR conditions were used to replicate the partial 16S ribosomal RNA (rRNA) sequence, using the RA20F2 (5ʹ-CAGCTTAACTGTAGAACTGC-3ʹ) and RA20R4 (5ʹ-TCGAGATTTGCATCACTTCG-3ʹ) primers described by Tsai *et al*. (32), in order to obtain a fragment suitable for phylogenetic analysis. The final product size was 662 bp.

### Sequencing and phylogenetic analysis

Amplicons of partial 16S rRNA were sequenced using the Sanger method in a commercial laboratory (Genomed, Warsaw, Poland) and used to create a phylogenetic tree. Sequencing was performed bidirectionally with forward and reverse primers. Chromatograms were analysed and quality-checked using FinchTV software (Geospiza, now Revvity Signals, Waltham, MA, USA), and then sequences were assembled with SeqTrace v. 0.9.0 (28). The assembled sequences were analysed in the EzBioCloud identification tool (5). The *Riemerella anatipestifer* DSM (Deutsche Sammlung von Mikroorganismen) 15868 type strain and two type strains closely related to RA, namely *Riemerella columbina* DSM 16469 and *Riemerella columbipharyngis* DSM 24015, were added to the study. All sequences obtained from the NCBI GenBank database were described with accession number, taxonomic name and DSM strain collection number. The alignment was performed using the ClustalW algorithm implemented in MEGA12 software (13), and the gapped columns were removed. The phylogenetic tree was created based on the maximum likelihood method and the Tamura–Nei model of nucleotide substitutions (30). The analytical procedure encompassed 27 nucleotide sequences with 556 positions in the final dataset. To compute the evolutionary divergence between sequences, the maximum composite likelihood model was used with the pairwise deletion option for all ambiguous positions for each sequence pair (31). The strains examined in this study were deposited in the NCBI GenBank database with accession Nos PX239683–PX239706.

### Antibiotic susceptibility testing

Antimicrobial susceptibility testing (AST) was performed using the disc diffusion (Kirby–Bauer) method with 14 antibiotics used in veterinary medicine (Oxoid, Basingstoke, UK): ampicillin (AMP, 10 μg), penicillin G (P, 10 units), cephalexin (CL, 30 μg), ceftazidime (CAZ, 30 μg), cefuroxime (CFX, 30 μg), amikacin (AK, 30 μg), streptomycin (S, 25 μg), clindamycin (DA, 2 μg), spectinomycin (SH, 100 μg), erythromycin (E, 30 μg), doxycycline (DO, 30 μg), enrofloxacin (ENR, 5 μg), chloramphenicol (C, 30 μg) and sulfamethoxazole + trimethoprim (SXT, 25 μg). Single colonies of each strain were dissolved in sterile 0.85% saline to assess turbidity at 0.5 in the McFarland standard using a DEN-1 densitometer (Biosan, Riga, Latvia). Bacterial suspension was streaked onto Mueller–Hinton agar with 5% defibrinated sheep blood (Oxoid) and incubated for 24 h at 37°C in a candle jar. Then, the inhibition zone diameters were measured. *Streptococcus pneumoniae* ATCC 49619 was used as a quality-control organism and incubated in conditions recommended by the Clinical and Laboratory Standards Institute (7). Because no interpretive criteria for RA have been set down in international standards, neither by the Clinical and Laboratory Standards Institute nor by the European Committee on Antimicrobial Susceptibility Testing, the results were recorded as the distribution of inhibition zone diameters (IZDs) for each antimicrobial.

### Phytobiotic susceptibility testing

The broth microdilution method was used to evaluate the susceptibility to *trans*-cinnamaldehyde, carvacrol, eugenol and geraniol. Phytochemicals and DMSO were purchased from Sigma-Aldrich (St. Louis, MO, USA). Stock solutions of each phytochemical dissolved in DMSO were prepared at a concentration of 30 mg/mL, and working solutions were prepared from this concentration at 3,000 μg/mL, 1,500 μg/mL, 750 μg/mL, 375 μg/mL and 187.5 μg/mL. The working solutions consisted of TSB (Biomaxima, Warsaw, Poland) and DMSO with the active substance (AS). To exclude the influence of DMSO on bacterial viability, control reactions were performed without ASs at DMSO concentrations of 1.25%, 2.5% and 5% (v/v). The inhibitory effect of DMSO on all RA strains included in this study was reached at the 5% concentration (v/v), and the DMSO concentration in the test wells never exceeded 1% (v/v). The minimum inhibitory concentration (MIC) and minimum bactericidal concentration (MBC) were defined using 96-well microtitre plates. Tryptic soy broth was inoculated with RA and incubated overnight. Following incubation, the turbidity was assessed with a DEN-1 densitometer (Biosan) and adjusted to 0.5 McFarland by adding sterile TSB. A 10-μL aliquot of the RA-inoculated TSB was transferred into a well containing 170 μL of TSB and 20 μL of the working solution of AS. Positive (190 μL of TSB + 10 μL of RA-inoculated TSB), negative (200 μL of TSB) and DMSO (170 μL of TSB + 10 μL of RA-inoculated TSB + 20 μL of DMSO solution) controls were included. All reactions were performed in triplicate. The sealed plates were incubated at 37°C for 24 h. After incubation, 5 μL from negative or suspected wells was transferred into a new microtitre plate with 195 μL of TSB in each well to evaluate the MBC, which is defined as the concentration of antimicrobial that effectively inactivates 99.99% of the bacteria present. Resazurin solution (Sigma-Aldrich, St. Louis, MO, USA) 0.015% was used as a bacterial growth indicator. The results were noted as the distribution of MIC and MBC values for each phytochemical, as well as MIC_50_/MBC_50_ and MIC_90_/MBC_90_, representing the concentrations inhibitory or bactericidal to 50% and 90% of the strains, respectively.

## Results

### Identification

All the strains exhibited similar morphology on blood agar. After 48 h of incubation, single colonies were round, greyish and mucoid, with a tendency to coalesce. All the strains were confirmed to be catalase and oxidase positive. After Gram staining, small Gram-negative rods were observed. Electrophoresis of the RA-specific PCR products confirmed the presence of the 338-bp product for each strain. The sequences of the partial 16S rRNA of the strains showed 99.28–100% similarity to that of the RA DSM 15868 type strain.

### Phylogeny

**Fig. 1.**
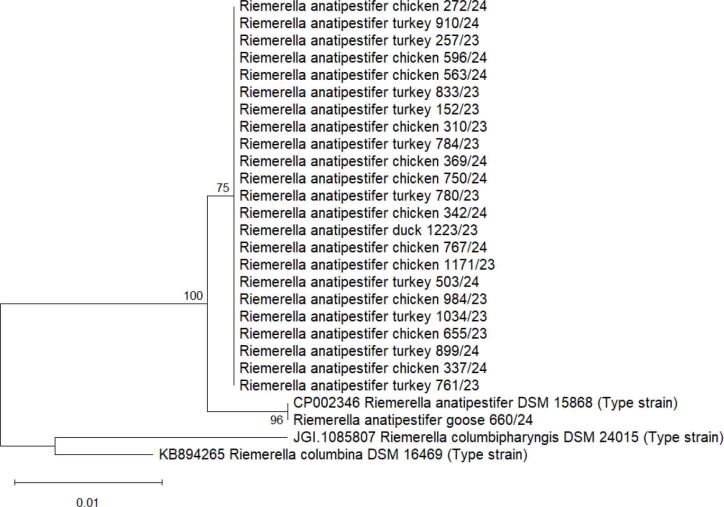
Maximum likelihood phylogenetic tree of *Riemerella anatipestifer* and closely related strains based on the partial 16S rRNA sequence. Bootstrap support values are shown next to the branches

The phylogenetic tree consisted of two main clusters (Fig. 1). The basal cluster contained two type strains closely related to RA, namely *Riemerella columbina* and *Riemerella columbipharyngis*. The second cluster was formed by the RA strains. These included the partial 16S rRNA sequences of the field isolates which were the object of our study, and the *Riemerella anatipestifer* DSM 15868 type strain sequence. All the partial RA 16S rRNA sequences included in the phylogenetic tree showed similarity over 99.2%. Three nodes were formed in the phylogenetic tree, with the most abundant group comprising 23 strains. The majority of sequences obtained in our study were identical and clustered together; however, strain 660/24, isolated from a goose flock, clustered separately with the *Riemerella anatipestifer* DSM 15868 type strain, as their sequences showed 100% identity.

### Antibiotic susceptibility

For nearly every antimicrobial, the IZD distribution was quite wide. The results are shown in Tables 2a and b. For AMP, P and CFX, the IZDs ranged from the lowest (<10 mm) to the highest it was possible to measure (>40 mm). However, the results usually fell near a finite value. For DA and E, the results were highly repetitive, and no zones of inhibition were observed, although a few strains were inhibited over a large IZD. For AMP, P and SH, the most common IZD was 30 mm. For CL, CAZ and CFX, in most cases it equalled or exceeded 40 mm. For AK and C, the IZDs were mainly 20 mm. Enrofloxacin and S, together with DA and E, formed no visible IZD around the disc in more than 70% of cases, so the IZDs were recorded as <10 mm. The most common IZD for SXT was 34 mm.

**Table 2a.** Distribution of <25mm inhibition zone diameters of *Riemerella anatipestifer* under the action of selected antibiotics

AM	Number of strains inhibited in a zone of the stated diameter (diameters in mm)																
	<10	10	11	12	13	14	15	16	17	18	19	20	21	22	23	24	25
AMP	1		1														2
P	1		1														3
CL							1										
CAZ												1					
CFX	1																
AK									1	1	3	8	2	3	1	2	
S	17						3			1		1		1	1		
DA	22																
SH											1			3	2		1
E	21											1					
DO							1		4	1		2		5	3	1	4
ENR	18			2						2		2					
C							1		1	3	3	8	3	1		1	1
SXT																	1

**Table 2b.** Distribution of >25mm inhibition zone diameters of *Riemerella anatipestifer* under the action of selected antibiotics

AM	Number of strains inhibited in a zone of the stated diameter (diameters in mm)															
	26	27	28	29	30	31	32	33	34	35	36	37	38	39	40	>40
AMP			1	1	7	1	1	2			1				5	1
P		2		1	7		2	1	2				2			2
CL								1		1	1	1	1	1	11	6
CAZ					2				1		2	1	1	2	10	4
CFX									1	1	1	1			8	11
AK	1	1	1													
S																
DA					1						1					
SH	2	2	3	1	7	1			1							
E		1													1	
DO	1				2											
ENR																
C	1								1							
SXT	1	1	1		3	1	4		6		1				3	2

1 AM – antimicrobial; AMP – ampicillin; P – penicillin G; CL – cephalexin; CAZ – ceftazidime; CFX – cefuroxime; AK – amikacin; S – streptomycin; DA – clindamycin; SH – spectinomycin; E – erythromycin; DO – doxycycline; ENR – enrofloxacin; C – chloramphenicol; SXT – sulfamethoxazole + trimethoprim

### Phytobiotic susceptibility

The phytochemical substance with the lowest MIC was *trans*-cinnamaldehyde. Nine strains exhibited susceptibility to this phytochemical at the 37.5 μg/mL concentration; however, the MIC_50_ and MIC_90_ for *trans*-cinnamaldehyde was 75 μg/mL. Geraniol was the second-most-effective substance against RA strains, with MIC_50_ and MIC_90_ values of 75 and 150 μg/mL, respectively. For carvacrol, both the MIC_50_ and MIC_90_ were 150 μg/mL, and for eugenol, both values were 300 μg/mL. One RA strain grew in every eugenol concentration examined; therefore, the MIC for the strain was shown as >300 μg/mL. Not a single strain was inhibited by the concentration of 18.75 μg/mL, regardless of the phytochemical. Detailed data are provided in Table 3.

**Table 3.** Distribution of minimum inhibitory concentration (MIC) of selected phytogenics for *Riemerella anatipestifer* strains

Phytogenic	Number of strains susceptible to this MIC (MIC in μg/mL)						MIC_50_ μg/mL	MIC_90_ μg/mL
	>300	300	150	75	37.5	18.75		
*Trans*-cinnamaldehyde				15	9		75	75
Geraniol		1	8	15			75	150
Eugenol	1	17	6				300	300
Carvacrol		1	17	6			150	150

The MBC_50_ and MBC_90_ of *trans*-cinnamaldehyde against RA in most cases was 75 μg/mL. Those of geraniol were assessed as 75 μg/mL and 150 μg/mL, respectively. For carvacrol, the MBC_50_ was150 μg/mL and the MBC_90_ 300 μg/mL, and it was for eugenol that the highest MBC_50_ and MBC_90_ were noted, both being 300 μg/mL. Summarised information can be found in Table 4.

**Table 4.** Distribution of minimum bactericidal concentration (MBC) of selected phytogenics for *Riemerella anatipestifer* strains

Phytogenic	Number of strains susceptible to this MBC (MBC in μg/mL)						MBC_50_ μg/mL	MBC_90_ μg/mL
	>300	300	150	75	37.5	18.75		
*Trans*-cinnamaldehyde			1	22	1		75	75
Geraniol	1		19	4			150	150
Eugenol	1	22	1				300	300
Carvacrol		4	20				150	300

## Discussion

Riemerellosis is a serious septicaemic poultry disease primarily associated with waterfowl, and numerous studies of it originate from countries with large waterfowl production. However, in recent years, an increased number of studies have been published focusing on RA occurrence in poultry species other than waterfowl, such as turkeys (16) and chickens (34). In our study, we performed phylogenetic analysis based on the partial 16S rRNA sequence to investigate the relationship between RA isolates in flocks of chickens, turkeys, geese and ducks. All the RA sequences included in the phylogenetic tree showed relatively low genetic diversity, as the similarity never fell below 99.2%, and 23 out of 24 field strains included in our study were identical. Similar results were shown by Nowaczek *et al*. (19) in a phylogenetic tree based on the *rpo*B gene sequence, where most RA field isolates clustered together. Although it should be confirmed by a larger sample size, this finding is evidence of the paucity of strains circulating in commercial Polish poultry flocks. It suggests with high probability that RA transmission is likely due to inadequate biosecurity on farms, such as through improper poultry manure handling, wild animal access and insufficient disinfection efforts. Furthermore, according to the phylogenetic tree, it emerged that closely related RA isolates can infect various poultry species, indicating that a farm that keeps one poultry species can serve as a source of infection for another keeping a second species. A similar conclusion was drawn by Sawicka-Durkalec *et al*. (26) in their study, which investigated the prevalence and phylogenetic analysis of RA strains among commercial and wild bird populations in Poland.

Antibiotic resistance is a serious threat that has been growing uncontrollably over the last decades. The major reason for the disquiet felt about it is its significance for human medicine and public health. Some pathogens can easily cross the borders between animal and human hosts, posing a threat to both and contributing to the spread of antimicrobial resistance. For this reason, it is strongly recommended to reduce and, where possible, exclude the use of specified antibiotics in veterinary medicine, especially in livestock, and to find alternatives to them. Worsening antibiotic resistance is seen extensively in veterinary medicine. Usually, research attention is focused on resistance acquisition by pathogens that exhibit zoonotic potential and threaten public health, such as *Salmonella, Campylobacter, Yersinia* and *Listeria*. However, similar research is needed on bacteria pathogenic only to animals, such as RA, as they are a significant reason for increased mortality and decreased welfare. For the majority of RA strains investigated in this study, non-visible IZD (<10 mm) were present in susceptibility testing to erythromycin, clindamycin, streptomycin and enrofloxacin. Although no interpretive criteria exist for RA, the lack of any inhibition zone could be considered demonstrative of resistance. These results are in agreement with the results obtained by Gyuris *et al*. (11), who demonstrated RA’s high resistance to streptomycin, gentamycin and erythromycin, as well as high resistance to flumequine (a first-generation quinolone) and intermediate susceptibility and resistance of a significant percentage of strains to enrofloxacin (86 % strains in total). The authors specified the breakpoints used in their study, and overlaying them on ours, the present results were also similar with regard to penicillin, doxycycline, spectinomycin and sulfamethoxazole with trimethoprim. In terms of resistance to amikacin, Nowaczek *et al*. (19) reported a large number of resistant RA strains, with MIC_50_ and MIC_90_ values of 128 μg/mL. On the other hand, in our study, as well as in the study conducted by Zhong *et al*. (39), amikacin resistance was not very common among RA strains. Those authors reported that only 9.5% of strains were resistant, and the 15–16 mm of IZD breakpoint used for amikacin by them classifies all our strains as susceptible. One probable reasons for this discrepancy is the different diagnostic methods used for the evaluation of antimicrobial susceptibility, and another is the unknown and possibly very different histories of antibiotic use on the farms which were sampled, as bacteria can acquire a resistance phenotype through the overuse of antibiotics.

Despite the listing of fluoroquinolones in antibiotic category B (use of which is to be restricted) by the European Medicines Agency (9), they are still some of the most commonly used antibiotics in veterinary medicine in Poland (8). In 1997, a study conducted by German scientists proved that enrofloxacin was effective against RA-induced septicaemia in ducklings (33). However, similarly to how our findings did not validate this, most later studies also did not, as many authors attested to high resistance levels to enrofloxacin in RA (11, 40). This progression from susceptibility to resistance over time underscores the growing level of antibiotic resistance and the urgency of minimising the use of antibiotics, particularly those of critical importance to human medicine. Similarly to fluoroquinolones, penicillins (especially aminopenicillins) and cephalosporins are crucial classes of antimicrobials used in veterinary medicine, with their activity spectrum encompassing a wide range of Gram-negative and Gram-positive organisms. The results of AST obtained in our study showed that penicillins and cephalosporins mostly produced a large IZD for RA. There is an inconclusiveness regarding susceptibility to these classes of antimicrobials of RA in existing research. On the one hand, researchers have claimed that the majority of RA strains were susceptible to penicillins and cephalosporins, proving that these antimicrobials are effective in riemerellosis treatment (17, 29). On the other hand, some authors have reported numerous RA strains resistant to penicillins and/or cephalosporins (6, 39). Here too, the potential reasons for those divergences are different diagnostic methods and the previous antibiotic use patterns on the farms rearing the birds from which RA was isolated – extremes of difference in their use cannot be excluded where there was no anamnesis taken. However, it appears that the most significant factor is the use of different interpretive criteria for susceptibility, which is exemplified by the breakpoint for ampicillin by Chinese researchers having been 14–16 mm (39) while that used by Hungarian researchers was 24 mm (11). As is seen, standardised interpretive criteria are needed to avoid confusion, as well as to provide proper information about strain susceptibility and make easier selection of the best antimicrobial therapy.

Unfortunately, years of overuse of antimicrobials, such as the practice of feeding animals subtherapeutic doses of antibiotics for growth promotion, have led to the selection of drug-resistant bacteria and their uncontrolled spread in the environment (3). To avoid the expansion of this phenomenon, some alternatives have been proposed, including the use of essential oils and natural substances derived from plants. In our study, besides the evaluation of antibiotic susceptibility, we established the efficacy of the antimicrobial properties of four phytogenics, namely *trans*-cinnamaldehyde, carvacrol, eugenol and geraniol, against RA. Their order of effectiveness from lowest MIC and MBC against RA to highest was *trans*-cinnamaldehyde, geraniol, carvacrol and eugenol. In a previous study, the antimicrobial properties of essential oils against veterinary pathogens were evaluated. LeBel *et al*. in Canada (14) studied the susceptibility of the six most common porcine respiratory bacterial pathogens: *Streptococcus suis, Actinobacillus pleuropneumoniae, Pasteurella multocida, Bordetella bronchiseptica, Haemophilus parasuis* (*Glaesserella parasuis*) and *Actinobacillus suis* to essential oils. They found that cinnamon (containing *trans-*cinnamaldehyde), thyme (a thymol source) and winter savory oils (with carvacrol as a constituent) were the most effective. Other Canadian scientists also evaluated the antibacterial activity of *trans*-cinnamaldehyde and eugenol against bovine respiratory pathogens (21). The results obtained by them correspond to our findings: for example, the susceptibility of the *Pasteurella multocida* ATCC 43137 strain (the most closely related to *Riemerella anatipestifer* amongst the bacteria included in their study) to *trans*-cinnamaldehyde and eugenol was noted with the phytogenics at 62.5 and 250 μg/mL concentrations, respectively, and differed little from the susceptibility to the same substances at 75 and 300 μg/mL in our study. Relatively low MICs were also obtained for *Mycoplasma bovis, Mannheimia haemolytica* and *Streptococcus dysgalactiae*. Van *et al*. (35) investigated the susceptibility of common poultry bacterial pathogens to various essential oils, including those derived from cinnamon and oregano. In their work, cinnamon essential oil transpired to be the most effective among the examined substances against every studied pathogen, and the MIC_50_ values were 0.3 mg/mL for *Escherichia coli* and *Gallibacterium anatis*, 1 mg/mL for *Avibacterium endocarditidis* and *Ornithobacterium rhinotracheale* and 2 mg/mL for *Pasteurella multocida*. Their results are in line with ours, indicating that *trans*-cinnamaldehyde, which constitutes the larger part of cinnamon essential oil’s active substances, possesses one of the strongest antibacterial activities. Research also supports the utility of another phytogenic besides *trans-*cinnamaldehyde: Chinese scientists demonstrated in a broiler infection model that encapsulated carvacrol significantly reduced the presence of *Clostridium perfringens* in the intestines, thereby preventing the development of necrotic enteritis in chickens and eliminating the need for antibiotic use for this purpose (15). The use of phytochemicals as alternatives to antibiotics in animal production is promising; however, more data are needed, especially in terms of pharmacokinetics and pharmacodynamics.

## Conclusion

The research reveals the relatively low genetic diversity of RA strains circulating in Polish poultry flocks but highlights the dynamic changes in antibiotic susceptibility among these strains. It also emphasises the need for continuous monitoring of antimicrobial susceptibility and underlines the urgency of establishing standardised breakpoints for AST interpretation in relation to *Riemerella anatipestifer*. This report joins others positing phytogenic substances as promising alternatives to antibiotics with relatively low MICs and MBCs; however, further research is needed in terms of these substances’ possible utilisation in the poultry industry, including species-specific differences in their disposition and pharmacological activity.
